# Effect of Acute and Chronic Administration of Carbamazepine on Cisplatin-Induced Hyperalgesia in Rats

**DOI:** 10.17795/jjnpp-3559

**Published:** 2012-01-04

**Authors:** Alireza Mohajjel Nayebi, Hamdollah Sharifi, Mohammad Ramadzani, Hassan Rezazadeh

**Affiliations:** 1 Drug Applied Research Center, Tabriz University of Medical Sciences, Tabriz, IR Iran; 2 Department of Pharmacology and Toxicology, Fcaulty of Pharmacy, Tabriz University of Medical Sciences, Tabriz, IR Iran

**Keywords:** Cisplatin, Carbamazepine, Neuralgia

## Abstract

**Background:**

Cisplatin is an effective antineoplastic drug used extensively in the treatment of malignancies. It induces painful peripheral neuropathy at high doses.

**Objectives:**

The aim of this study was to investigate the effect of carbamazepine (CBZ) on cisplatin-induced peripheral neuropathic pain by using the tail-flick test.

**Materials and Methods:**

The study was performed using male Wistar rats weighing 180–200 g. Neuropathic pain was induced by intraperitoneal (IP) administration of cisplatin (5 mg/kg). The effect of oral (PO) CBZ administration (5, 10, and 15 mg/kg) on cisplatin-induced pain was assessed using the tail-flick test.

**Results:**

Our results showed that cisplatin (5 mg/kg, IP) induced egregious pain (P < 0.01) on day 15. Acute administration of CBZ (5, 10, and 15 mg/kg, PO) caused significant (P < 0.05) increase in tail-flick time latency in a dose-dependent manner, in comparison with that observed in the control group. Furthermore, chronic administration of CBZ (5, 10, and 15 mg/kg, PO) increased (P < 0.05) the pain threshold on days 5 and 10. The analgesic effect of morphine (5 mg/kg, IP) was greater than that after acute CBZ administration (5, 10, and 15 mg/kg, PO).

**Conclusions:**

Our results showed that both acute and chronic CBZ administration attenuated cisplatin-induced pain. We suggest that CBZ can be used clinically for alleviating cisplatin-induced neuropathic pain in cancer patients, without any limitations such as tolerance to analgesic effect.

## 1. Background

Cisplatin is an effective antineoplastic drug used extensively in the treatment of malignancies, including ovarian, bladder, and testicular cancers. Cisplatin acts by cross-linking DNA and blocking its replication in rapidly dividing cells (1, 2). High doses of cisplatin during chemotherapy have long been known to induce painful peripheral neuropathy. After discontinuation of treatment, the signs progress for a period of about 4 months, followed by spontaneous amelioration; however, normalization does not occur (3). Pain intensity varies among cancer patients and depends on a patient’s pain sensitivity, the type of cancer, and tumor location (4). Opioids, nonsteroidal anti-inflammatory drugs (NSAIDs), corticosteroids, local anesthetics, antidepressants, and anticonvulsants are used either alone or in combination for relieving pain. These drugs induce major problems that cause some limitations in their chronic use. For example, opioids may cause sedation, respiratory depression, and decrease in gastrointestinal motility; and NSAIDs can interfere with coagulation pathways or cause gastric ulcers and renal toxicity (4).

Carbamazepine (CBZ) is a major antiepileptic drug used for treating partial seizures and generalized tonic–clonic seizures. The main mechanism of action of CBZ is blocking sodium channels during rapid, repetitive, sustained neuronal firing and preventing post-tetanic potentiation (5). CBZ is effective in relieving chronic pain caused by damage (due to injury or disease) to nerves. The type of pain that responds well to CBZ is neuropathic pain (e.g., trigeminal neuralgia) (6) and painful diabetic complications (7).

## 2. Objectives

Our aim was to investigate the effect of acute (single dose) and chronic administration of CBZ on cisplatin-induced neuropathic pain in rats. Moreover, we compared the analgesic effect of CBZ and morphine (the standard analgesic drug) on neuropathic pain induced by cisplatin.

## 3. Materials and Methods

### 3.1. Animals

The study was performed using 40 male Wistar rats weighing 180–200 g. The rats were divided randomly into equal groups (n = 8 per group) and were kept in standard Plexiglas cages (4 animals per cage) at room temperature (21 ± 3 °C) and a 12-h light-dark cycle. All experiments were performed according to the ethical guidelines of the Tabriz University of Medical Sciences for the care and use of laboratory animals.

### 3.2. Drugs

All the drugs were purchased from Sigma, USA, except for cisplatin, which was purchased from EBEWE Pharma, Austria. Cisplatin solution was freshly prepared on the day of the experiment by dissolving in 0.9% saline, and intraperitoneal (IP) administration of cisplatin was performed. CBZ was dispersed in glycerin oil, and oral (PO) administration through an oral gavage needle at doses of 5, 10, and 15 mg/kg was performed.

### 3.3. Induction of Hyperalgesia

IP administration of 5 mg/kg of cisplatin was performed to induce hyperalgesia. Then, the pain threshold was assessed every day by using the tail-flick test, until significant hyperalgesia was observed.

### 3.4. Tail Flick Test

The tail-flick assay was used for measuring nociceptive sensitivity after acute and chronic CBZ administration in rats. The nociceptive stimulus was applied to the distal 4 cm of the tail by using a tail-flick unit (Mod.37360, UGO BASILE, Italy) with an irradiation intensity of 20 mW/cm2. Forty seconds was considered as the cutoff time to avoid further tissue damage. The latency time (s) for withdrawal of the tail from the heat-producing beam of the apparatus was recorded at the end of the experiment. The tail-flick test was performed 1 h and 30 min after PO and IP administration of the drugs, respectively.

### 3.5. Statistical analysis

Comparisons between each data set were calculated using the SPSS software (version 16). The data were expressed as mean ± SEM values and were analyzed using one-way analysis of variance. A P value <0.05 was considered statistically significant. In case of significant variation (P < 0.05), the values were compared using the Tukey test.

## 4. Results

**Figure 1:**
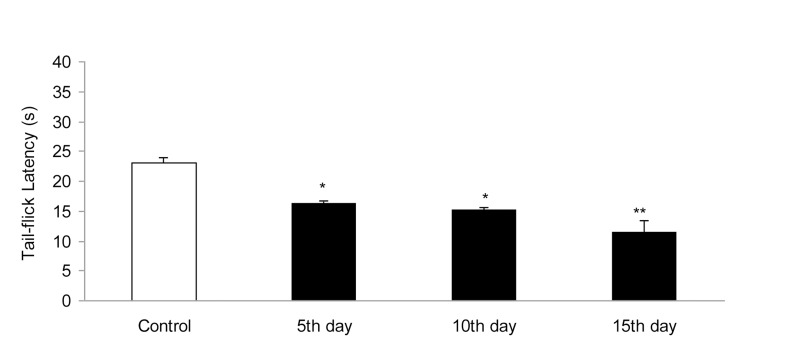
Effect of Cisplatin (5 mg/kg, IP) on Tail-Flick Latency Time (s). Data has been Shown as Mean ± SEM Values; n = 8 Rats per Group; ANOVA Followed by the Tukey Test. *P < 0.05 and **P < 0.01, when Compared with the Control Group.

**Figure 2:**
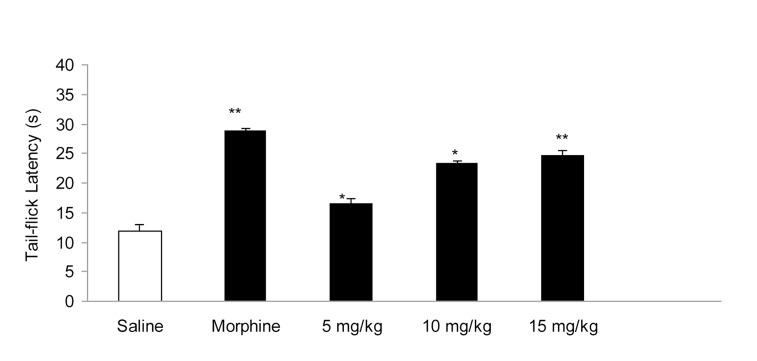
Effect of Acute Administration of Morphine (5 mg/kg, IP) and Carbamazepine (5, 10, and 15 mg/kg, PO) on Cisplatin (5 mg/kg, IP)- Induced Pain. Data has been Shown as Mean ± SEM Values; n = 8 Rats per Group; ANOVA Followed by the Tukey Test. * P < 0.05 and * P < 0.01, when Compared with the Saline-treated Group.

**Figure 3:**
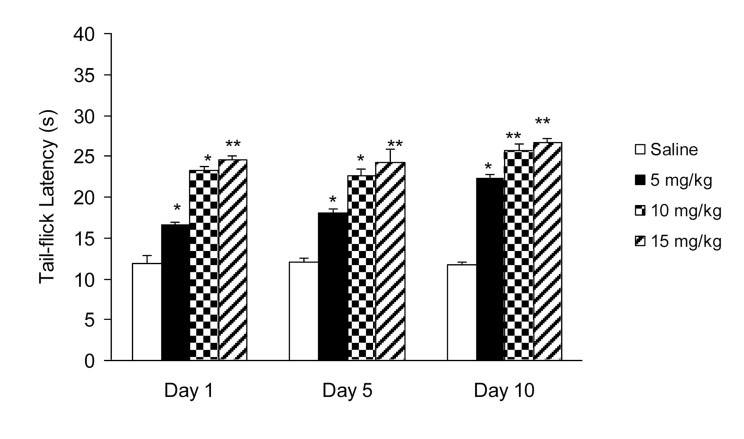
Effect of Chronic Administration of Carbamazepine (5, 10, and 15 mg/kg, PO) on Cisplatin (5 mg/kg, IP)-Induced Pain. Data has been Shown as Mean ± SEM Values; n = 8 Rats per Group; ANOVA Followed by the Tukey Test. * P < 0.05 and ** P < 0.01 when Compared with the Saline-treated Group.

IP administration of cisplatin (5 mg/kg) produced significant (P < 0.01) hyperalgesia on day 15 (Figure 1). This dose of cisplatin was able to induce neuropathic pain in rats. Oral gavaged CBZ at single doses of 5, 10, and 15 mg/kg resulted (P < 0.01 and 0.05) in increase in tail-flick latency time. The greatest analgesic effect, which was less than the analgesic effect of morphine (5 mg/kg, IP), was observed at the dose of 15 mg/kg (Figure 2). There was no decrease in the analgesic effects of CBZ (5, 10, and 15 mg/kg, PO) during chronic treatment (Figure 3). Both prolonged and acute (single dose) administration of CBZ (5, 10, and 15 mg/kg, PO) resulted in similar decrease in cisplatin-induced hyperalgesia.

## 5. Discussion

Many cancer chemotherapy treatments are limited by dose-related peripheral nervous system toxicity, a small-fiber painful peripheral neuropathy (8). Cisplatin is a frequently used chemotherapeutic agent that causes symptoms of neuropathy such as numbness, tingling, or burning in the extremities at higher doses (9). After discontinuation of treatment, the signs progress for a period of about 4 months, followed by spontaneous amelioration; however, normalization does not occur (3). In an animal model designed to mimic the condition in human patients, animals showed mechanical allodynia and hyperalgesia that lasted as long as 15 days after cisplatin administration (4). In our study, cisplatin (5 mg/kg, IP) induced noticeable hyperalgesia on day 15. Thereafter, this dose of cisplatin was used for inducing hyperalgesia during the experiments. This is in accordance with a previous study that reported hyperalgesia and neuropathic pain induced by cisplatin (10).

Several anticonvulsant drugs have emerged as being efficacious in treating some persistent pain syndromes and as alternatives to opioids and non-steroidal anti-inflammatory and tricyclic antidepressant drugs used for treating persistent pain. In particular, CBZ (sodium channel blocker) is efficacious in the treatment of trigeminal neuralgia and diabetic neuropathy (11). Because CBZ is administered orally, in this study, we treated rats with acute and chronic PO doses of CBZ (5, 10, and 15 mg/kg). Our results showed that acute CBZ administration induced pain-relieving effect in cisplatin-treated rats in a dose-dependent manner. The greatest analgesic effect was observed at the dose of 15 mg/kg. However, the analgesic effect of acute CBZ administration was less than that of morphine. This confirms the results of a previous study that reported the analgesic effect of CBZ in chronic neuropathic pain (12). The inhibition of voltage-gated sodium channels in the brain, inhibitory modulation of pain transmission at central adrenergic a-receptors by gama amino-butyric acid, and activation of peripheral adrenergic a-receptors are presumed to participate in the antinociceptive action of CBZ (5).

Furthermore, we evaluated the effect of chronic CBZ administration on cisplatin-induced hyperalgesia. We observed that chronic CBZ administration ameliorated cisplatin-induced pain in a dose-dependent manner. There was no significant alteration in the pain-relieving effects of CBZ during chronic administration; whereas, tolerance to analgesic effect is the greatest complication of opioids such as morphine that restricts their efficacy in long-term therapy. The analgesic effects of CBZ did not decrease with continuous therapy; therefore, we suggest that CBZ may be used as a potential alternative therapy for attenuating cisplatin-induced hyperalgesia. In summary, complications caused by anticancer drugs, especially cisplatin-induced hyperalgesia, is a major and common problem in patients with malignancies who need to be carefully managed. Drugs available for the amelioration of neuropathic pain (e.g., morphine) have major problems such as tolerance to analgesic effects. CBZ may be used for relieving neuropathic pain induced by chemotherapeutic drugs.

On the basis of our results, it can be concluded that both acute and chronic administration of CBZ ameliorates cisplatin-induced hyperalgesia in a dose-dependent manner. Further clinical trials should be performed to evaluate the efficacy of CBZ in treating cisplatin-induced pain.
